# Brazilian green propolis prevent Alzheimer’s disease-like cognitive impairment induced by amyloid beta in mice

**DOI:** 10.1186/s12906-023-04247-7

**Published:** 2023-11-17

**Authors:** Takashi Ito, Tomomi Degawa, Nobuaki Okumura

**Affiliations:** Institute for Bee Products & Health Science, Yamada Bee Company, Inc, Okayama, Japan

**Keywords:** Alzheimer’s Disease, Astrocytes, Brazilian green propolis, Cognitive impairment, Microglia

## Abstract

**Background:**

The increasing incidence of cognitive impairment has become a health problem in the aging society. Owing to its antioxidant and anti-inflammatory properties, Brazilian green propolis—derived from *Baccharis dracunculifolia*—is anticipated to possess anticognitive properties. However, the preventive effect of Brazilian green propolis on cognitive impairment remains unexplained. This study aimed to investigate the effect of Brazilian green propolis on cognitive impairment using a mouse model of Alzheimer’s disease (AD) induced by intracerebroventricular injection of amyloid beta (Aβ)_25‒35_.

**Methods:**

Five-week-old male Slc:ddY mice were randomly divided into five groups (n = 8). The groups were pretreated with vehicle and propolis at a dose of 100, 300 and 900 mg/kg body weight for 8 days, then AD-like phenotypes were induced by intracerebroventricular (ICV) injection of Aβ_25‒35_. A sham operation group was set as the control. Memory and learning ability were measured at 7 to 8 days after ICV injection. Gene expression and histological studies were performed at the endpoint of the study.

**Results:**

In a passive avoidance test, the administration of Brazilian green propolis prevented the impairment of learning and memory function. Furthermore, comprehensive gene expression analysis in the hippocampus and forebrain cortex revealed that Brazilian green propolis suppressed Aβ_25–35_-induced inflammatory and immune responses. In particular, Brazilian green propolis prevented alterations in gene expressions of microglial and astrocytic markers such as *Trem2* and *Lcn2* induced by Aβ_25‒35_ injection, suggesting the suppression of excessive activation of glial cells in the brain. In addition, Brazilian green propolis suppressed the elevation of plasma interleukin (IL)-6 levels induced by Aβ_25‒35_ injection.

**Conclusions:**

The results suggest that the prophylactic administration of Brazilian green propolis has a preventive effect against AD by suppressing excessive inflammation and immune response in glial cells. To our knowledge, this study is the first to demonstrate that Brazilian green propolis may inhibit the hyperactivation of microglia and astrocytes as a mechanism of action to prevent AD. Thus, it is a promising ingredient for preventing AD-type dementia.

**Supplementary Information:**

The online version contains supplementary material available at 10.1186/s12906-023-04247-7.

## Background

The increasing incidence of dementia in the aging society accompanies a decline in healthy life expectancy and quality of life [1, 2]. In addition, it has become a social problem, resulting in an economic burden. Depending on the causes, dementia can be categorized as Alzheimer’s disease (AD), vascular dementia, or Lewy body disease. However, definitive treatments for dementia are still undeveloped. Among the potential treatment options, natural products and nutrients derived from dietary foods may prevent and retard the progression of dementia [3, 4].

Propolis is a resinous mixture obtained from honeybees from various plant sources, such as tree buds and sap and is traditionally used in alternative medicine globally [5, 6]. Among the different types of propolis, Brazilian green propolis (derived from *Baccharis dracunculifolia*) has neuroprotective effects due to its antioxidant properties [7, 8] and anti-inflammatory effects on microglia under hypoxic culture conditions [9]. Furthermore, studies in an animal model have shown that Brazilian green propolis suppresses cognitive dysfunction induced by hyperhomocysteinemia [10]. A recent study has shown that Brazilian green propolis reduces cognitive impairment in older patients with mild cognitive impairment living at high altitudes [11]. In addition, propolis improves cognitive function in healthy elderly who are aware of forgetfulness in daily life [12]. These clinical trial findings suggest that Brazilian green propolis is an anticognitive impairment agent. However, the preventive effects of Brazilian green propolis on AD and its mechanism of action have not been fully elucidated.

The pathological features of AD are senile plaques and neurofibrillary tangles. Amyloid beta (Aβ) is a component of senile plaques. In particular, Aβ_1−40_ and Aβ_1−42_ peptides are reported to be the main components of senile plaques. On the other hand, Aβ_25−35_ peptide has also been reported to be responsible for a cytotoxic and oxidative stress [13, 14]. It can induce neurotransmitter disorders and neuronal cell death [15, 16] and has been recognized as a primary factor in the “amyloid hypothesis” in AD pathology. Animal models developed by directly transporting Aβ to the brain [17], and a transgenic mouse model overexpressing amyloid precursor protein [18] have been widely used to explain AD pathology and develop therapeutic agents against it. The Aβ intracerebroventricular (ICV) injection model is commonly used to screen for drugs and materials with preventive effects against AD-type dementia caused by rapidly induced learning and memory impairment with neurodegeneration, as observed in patients with early AD [19–22].

Glial cells such as microglia and astrocytes are involved in the onset of Alzheimer’s disease [23]. Excessive activation of these glial cells is observed in AD patients [24]. Microglia are the brain’s resident macrophages and play synapse elimination and debris removal [25]. Trem2 is an immunoreceptor expressed on the surface of microglia, and its expression is high in AD patients [26]. Recent research has shown that Trem2 leads to an inflammatory signal and plays as an Aβ clearance receptor [27]. Astrocytes are primary glial cells in the brain. In AD patients, astrocytes accumulate around snail plaques. Lipocalin 2 (LCN2) is a secreted glycoprotein known as a marker of active astrocytes [28, 29]. Recently, plasma Lcn2 has been listed as a potential diagnostic biomarker for Alzheimer’s disease [30].

Using AD model mice, this study aimed to investigate the preventive effects and elucidate the mechanism of action of Brazilian green propolis on cognitive impairment, particularly in improving learning and memory, using microarray-based comprehensive gene expression analysis. We also addressed the effect of Brazilian green propolis on systemic inflammation.

## Methods

### Animal experiments

**Fig. 1:**
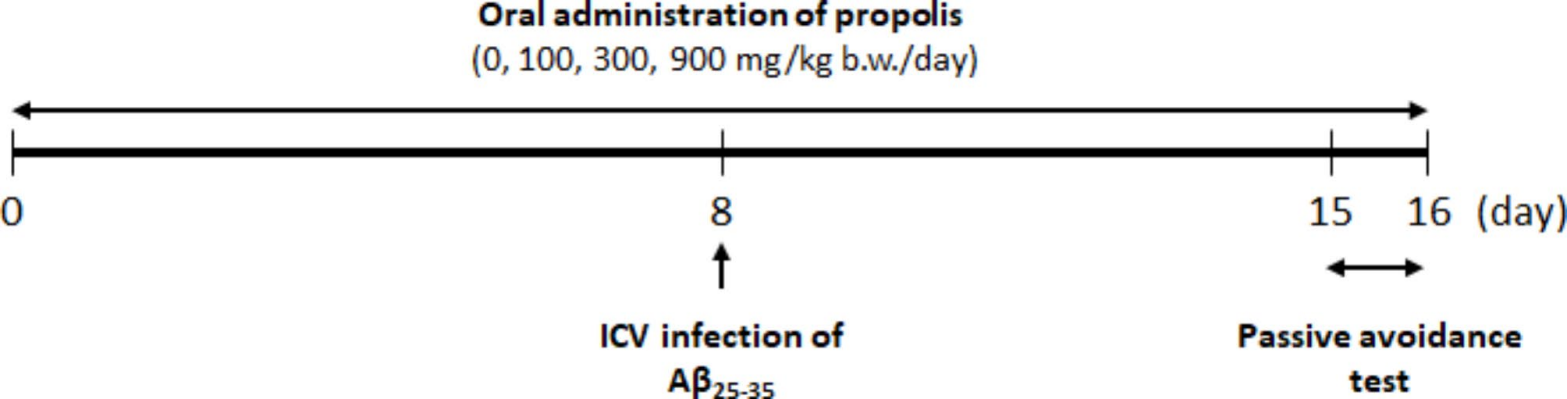
Experimental design of this study

All animal experiments were performed in accordance with the “Basic Guidelines for the Conduct of Animal Experiments in Implementing Agencies under the jurisdiction of the Ministry of Health, Labor, and Welfare, Japan” and approved by the Ethics Committee of Nihon Bioresearch Inc. (Gifu, Japan) based on their internal guidelines (Study No. 380,162) in accordance with ARRIVE guidelines. Five-week-old male Slc:ddY mice were obtained from Japan SLC, Inc. (Shizuoka, Japan) and acclimated for 1 week. Mice were housed in a plastic cage under a 12-h light/dark cycle maintained at a temperature range of 20‒26 °C and humidity of 40‒70%. The mice had *ad libitum* access to the MF diet (Oriental Yeast Co., Ltd., Tokyo, Japan) and water. The experimental design of these studies is shown in Fig. 1. The mice were randomly divided into five groups (n = 8 for each group) based on equal average body weight: sham [sham operation plus vehicle (0.5% methylcellulose) treatment], Aβ (ICV Aβ_25‒35_ injection plus vehicle treatment), Aβ + PP100 (ICV Aβ_25‒35_ injection plus 100 mg/kg b.w. Brazilian green propolis treatment), Aβ + PP300 (ICV Aβ_25‒35_ injection plus 300 mg/kg b.w. Brazilian green propolis treatment), and Aβ + PP900 (ICV Aβ_25‒35_ injection plus 900 mg/kg b.w. Brazilian green propolis treatment). The number of animals determined by refereeing previous studies [13, 22, 31] Brazilian green propolis was prepared using ethanol extraction followed by lyophilization. It was standardized to contain a minimum of 8% artepillin C and a minimum of 0.14% culifolin, which is a major cinnamic acid derivative. Powdered Brazilian green propolis (Lot. LY3-004) was then mixed with 0.5% methylcellulose and orally administered to the mice daily at 9‒12 a.m. We have chosen the dose of Brazilian green propolis in this study by referring previous studies [10, 32, 33]. Brazilian green propolis treatment started 8 days before ICV Aβ_25‒35_ injection and continued until the end of the experiments.

### ICV injection of Aβ_25-35_

We used ICV injection of Aβ_25‒35_ as a model of AD-like dementia [13, 20–22] to investigate the effect of Brazilian green propolis on learning and memory deficiency. In this model, Aβ_25‒35_ induces oxidative stress, inflammation, and neuronal damage in the cortex and hippocampus within 1–2 weeks, leading to AD-like dementia [14, 20]. After 8 days of Brazilian green propolis treatment, a single ICV injection of Aβ_25‒35_ was administered. Aβ_25‒35_ (PolyPeptide Laboratories, Limhamn, Sweden) was dissolved in 2 mM sterile distilled water, mixed thoroughly, and incubated at 37 °C for 4 days to obtain the aggregation form. Sterile distilled water was used for the sham group. After anesthetizing with 40 mg/kg sodium pentobarbital and fixing the mice, a stainless-steel pipe was inserted at a selected position, which was 1 mm lateral (right side) and 0.2 mm posterior from the bregma and 2.5 mm deep from the bone surface. ICV injection of Aβ_25‒35_ solution (6 nmol/3 µL) or water (3 µL) was administered using a microsyringe pump for 3 min. After implantation, the cranial hole was occluded with a non-absorbable bone marrow hemostatic agent (Nestop®, Alfresa Supply Service Co. Ltd., Osaka, Japan), and the scalp was sutured.

### Passive avoidance test

The passive avoidance test was conducted 7 days after ICV injection to assess learning and memory functions [20]. We used a step-through passive avoidance response device in a bright room (W: 100 × D: 100 × H: 300 mm), with a sliding guillotine door separating a dark room with an electrical shock device (W: 240 × D: 245 × H: 300 mm). For the acquisition trial (15 days of propolis administration), after 10 s of placing each mouse in the bright room, the guillotine door was opened. The time a mouse took to enter the dark room was recorded. Following this, the guillotine door was closed, and the mouse received an electrical stimulation (0.2 mA, 2s, scrambled method). For the test trial (16 days of propolis administration), we placed a mouse in the bright room and recorded the time it took to enter the dark room up to 300 s. After the test trial was completed, blood and tissue samples were collected under anesthesia with the inhalation of isoflurane. Plasma was obtained using centrifugation (4 °C, 2150 ×*g*, 15 min). Their brain was excised after further blood collection. The excised brains were placed in ice-cold saline, divided into the prefrontal cortex and hippocampus, and immediately frozen using liquid nitrogen. All samples were stored at − 80 °C until analysis.

### Microarray analysis

Total RNA from the prefrontal cortex and hippocampus was purified using the Nucleospin RNA Plus kit (Takara, Shiga, Japan), and its quality and quantity were confirmed using a bioanalyzer (Agilent Technologies, Santa Clara, CA, USA). Comprehensive gene expression analysis was performed using the Clariom S Mouse Array (Thermo Fisher Scientific, Waltham, MA, USA). Briefly, cRNA and single-strand (ss)-cDNA were synthesized from 100 ng total RNA using the GeneChip WT PLUS Reagent Kit. Then, ss-cDNA was fragmented, labeled, and hybridized to GeneChip using a hybridization cocktail of GeneChip Hybridization and Wash and Stain Kit (Thermo Fisher Scientific). Fluorescence was measured using the GeneChip Scanner 3000. Data were analyzed using Transcriptome Viewer (Kurabo, Japan). Gene Ontology analysis was performed by DAVID Bioinformatics Resources 6.8 (https://david.ncifcrf.gov/). The datasets were deposited in Gene Expression Omnibus (Accession number: GSE234109).

### Quantitative real-time polymerase chain reaction

Total RNA (500 ng) was reverse transcribed with the ReverTra Ace® quantitative real-time polymerase chain reaction (RT-qPCR) Master Mix (Toyobo, Osaka, Japan). RT-qPCR was performed using the SsoAdvanced™ Universal SYBR® Green Supermix (Bio-Rad Laboratories, Inc., Hercules, CA, USA). PCR primers used were as follows: Trem2 transcript variant 1 primers, 5′-TGCTGGAGGACCCTCTAGATGAC-3′ and 5′-CCCACAGGATGAAACCTGCCT-3′; Trem2 transcript variant 2 primers, 5′-ACAGCACCTCCAGGAATCAAGA-3′ and 5′-CACAGGTGTTCCCGGCTTCT-3′; and ACTB primers, 5′-TTCTTGGGTATGGAATCCTGTGGC-3′ and 5′-AGAGGTCTTTACGGATGTCAACG-3′.

### Immunofluorescence staining

The brain tissues were fixed with cold 4% paraformaldehyde (Nacalai Tesque Inc., Kyoto, Japan) solution for 16 h, ethanol was replaced with G-NOX (GenoStaff, Tokyo, Japan), and the tissues were paraffin-embedded. Section (10-µm thickness) were prepared using a microtome. The prepared sections were blocked with TNGS (0.3% Triton X-100, 1% normal goat serum) at room temperature (20 ± 2℃) for 1 h. Subsequently, an anti-TREM2 primary antibody (#91,068, Cell Signaling Technology (Danvers, MA, USA), rabbit, dilution 1:100) and an anti-IBA1 primary antibody (#17,198, Cell Signaling Technology, mouse, dilution 1:200) were incubated with the sections overnight at 4 °C. The sections were washed with phosphate-buffered saline with Tween 20 (T-PBS) and incubated with goat anti-rabbit immunoglobulin (Ig)G 555 (1:2000) and anti-mouse IgG 488 (1:2000) for 1 h at room temperature. After washing with T-PBS, the sections were stained with DAPI (4, 6- diaminido-2-phenylindole)-Fluoromount-G (Southern Biotech, Birmingham, AL, USA). Images were obtained and analyzed using a BZ-X800 microscope (Keyence, Osaka, Japan). Six sections were then selected from each mouse, and the cornu ammonis 1 (CA1) of the hippocampus was used for the analysis. Results showed the area of immunoreactive regions for TREM2 and Iba1.

### Measurement of plasma cytokine levels

Plasma interleukin (IL)-6, C-reactive protein (CRP), and transforming growth factor-β1 (TGFβ1) levels were measured using Luminex Multiplex Immunoassay (R&D Systems, Minneapolis, MN, USA).

### Statistical analyses

All statistical analyses were performed using GraphPad Prism7 (GraphPad Software, San Diego, CA, USA). We performed the Shapiro-Wilk test for a data normality. Statistical differences among groups were analyzed using one-way analysis of variance followed by Tukey’s test. If two groups were analyzed, the significance was determined using Student’s *t*-test. *P* < 0.05 was considered statistically significant.

## Results

### Effect of Brazilian green propolis on Aβ-induced learning and memory impairment

**Fig. 2:**
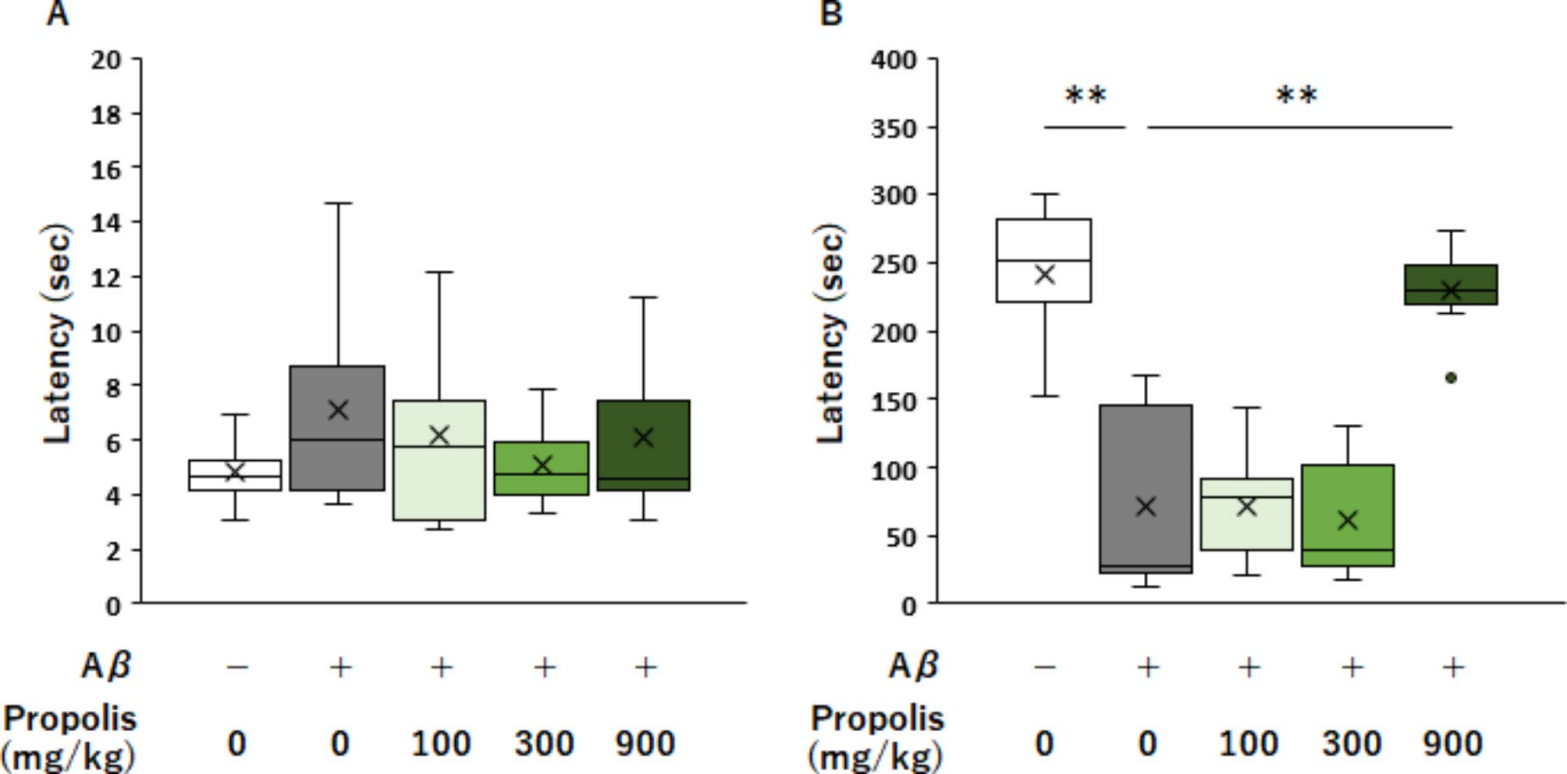
Effect of Brazilian green propolis on learning and memory impairment in the Aβ_25-35_-injected group. Latency time in the acquisition trial, n = 8 in each group (**A**). Latency time in the test trial, n = 8 in each group (**B**). Data are represented as boxplots show minimum, first-quartile, median, third-quartile, and maximum values. Outliers are shown as circles. *P*-values were determined using one-way analysis of variance (post-hoc test Tukey’s test), **P < 0.01

We first evaluated the effect of Brazilian green propolis on learning and memory impairment after ICV injection of Aβ_25‒35_ and a passive avoidance test. The latency time in the acquisition trial was similar among all the groups (Fig. 2A). The ICV injection of Aβ_25‒35_ significantly reduced latency time in the test groups compared with that in the sham group, suggesting the impairment of learning and memory abilities (Fig. 2B). The latency time among the test groups was similar to that in the Aβ group when 100 and 300 mg/kg/b.w. Brazilian green propolis were administered. However, the Aβ + PP900 group showed a significant increase in latency time to levels similar to those of the sham group compared with the Aβ group (Fig. 2B). Thus, pretreatment with Brazilian green propolis protected against Aβ-induced learning and memory impairment.

### Brazilian green propolis suppressed Aβ-induced changes in gene expression in the brain

**Fig. 3:**
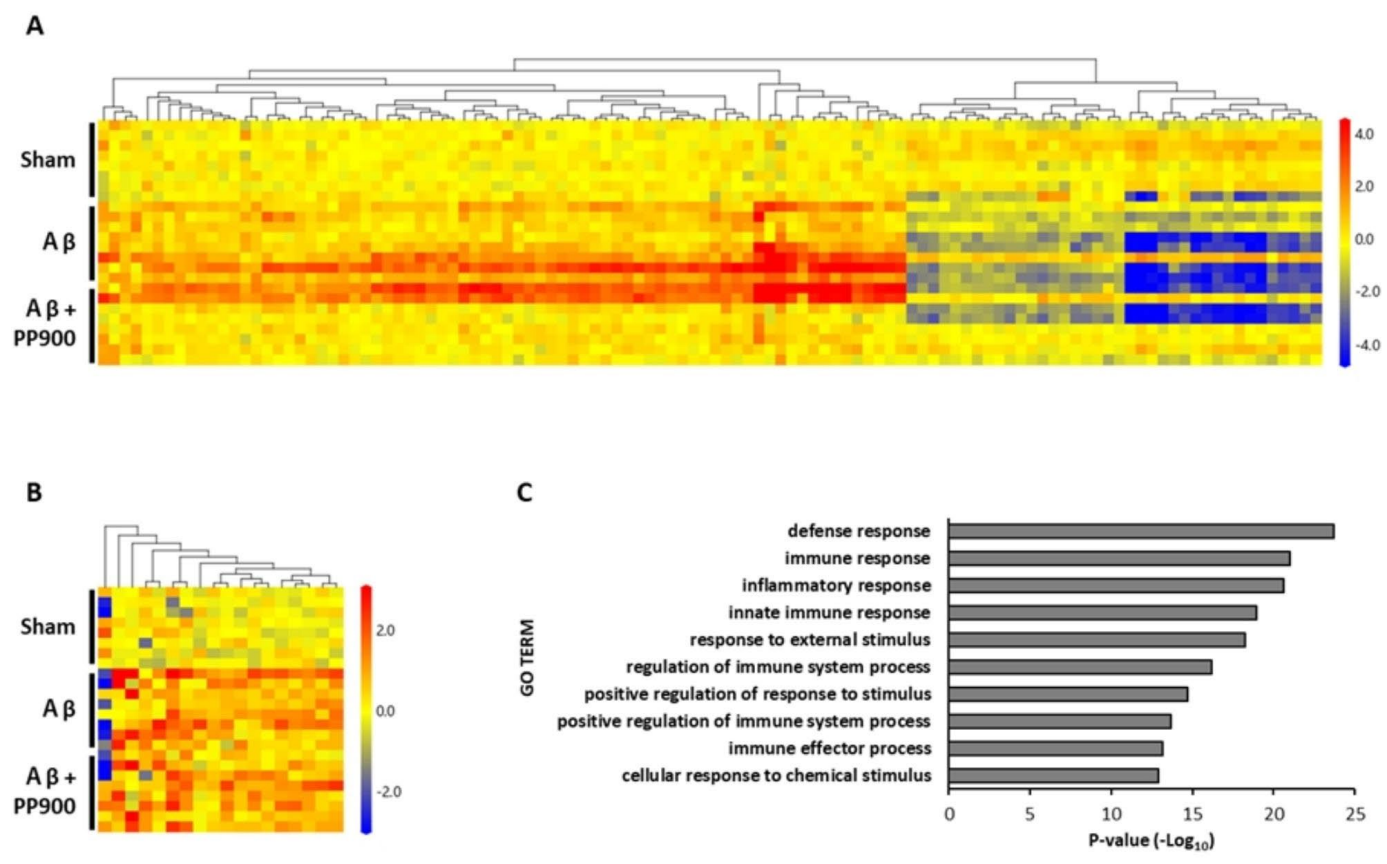
Changes in gene expression in the brain induced by Aβ_25-35_ injection. Heatmap analysis showed the transcripts that showed significant 1.8-fold (*P* < 0.05) change in the hippocampus (**A**) or prefrontal cortex (**B**) in the Aβ group compared with that in the sham group, n = 8 in each group. (**C**) Enrichment analysis of differentially expressed genes between the sham and Aβ groups in biological processes using DAVID.

The mechanism of Brazilian green propolis in preventing Aβ_25‒35_-induced learning and memory impairment was elucidated using comprehensive gene expression analysis in the hippocampus and prefrontal cortex. In the hippocampus, ICV Aβ_25‒35_ injection altered the expressions of 112 genes (Fig. 3A), including 74 upregulated genes (Additional Table 3) and 38 downregulated genes (Additional Table 2). In the prefrontal cortex, the expressions of 18 genes were altered (Fig. 3B), 17 of which were upregulated genes and 1 was downregulated (Additional Table 3). Additionally, the expressions of 12 genes changed in, both, the hippocampus and prefrontal cortex. Gene Ontology analysis using DAVID revealed that the differentially expressed genes in the hippocampus were mainly related to defense, inflammatory, and immune responses (Fig. 3C).

We further examined the effect of Brazilian green propolis treatment on alterations in the expressions of these genes. In the Brazilian green propolis groups, the Aβ-induced altered gene expressions tended to be suppressed (Additional Tables 1, 2 and 3). In particular, in the hippocampus, the genes *Lcn2*, *Trem2*, *Spp1*, *Cxcl13*, *Folr1*, and *Kcnj13* were significantly induced by > 2 folds after Aβ infusion and inhibited by > 2 folds after Brazilian green propolis administration (Table 1). Among the genes whose expressions in the prefrontal cortex were significantly altered by ICV Aβ_25‒35_ injection, *Trem2* and *Gbp3* were significantly suppressed by Brazilian green propolis treatment (Table 2).

**Table 1:** Genes suppressed by Brazilian green propolis treatment in the hippocampus

Gene Symbol	Gene Accession	Value (LOG_2_ Ratio)			Log Ratio ^1)^	*p*-value ^1)^	Log Ratio ^2)^	*p*-value ^2)^
		Sham	Aβ	Aβ + PP900				
*Lcn2*	NM_008491	3.8	8.0	6.2	4.13	< 0.001	−1.73	0.184
*Trem2*	NM_010821	7.5	10.4	9.8	2.93	0.002	−0.63	0.110
*Spp1*	NM_001204201	5.2	8.0	6.9	2.84	0.010	−1.15	0.457
*Cxcl13*	NM_011337	4.7	7.0	6.4	2.29	0.007	−0.57	0.131
*Kcnj13*	NM_001110227	14.0	10.1	11.08	−3.95	0.013	1.02	0.110
*Folr1*	NM_001252552	11.4	8.1	9.20	−3.29	0.040	1.09	0.184

^1)^ Log ratio between the sham and Aβ groups, *p*-value determined using Student’s *t*-test

^2)^ Log ratio between the Aβ and Aβ + PP900 groups, *p*-value determined using Student’s *t*-test

Sham group [sham operation plus vehicle (0.5% methylcellulose) treatment]; Aβ group (ICV Aβ_25‒35_ injection plus vehicle treatment); Aβ + PP900 group (ICV Aβ_25‒35_ injection plus 900 mg/kg b.w. Brazilian green propolis)

**Table 2:** Genes suppressed by Brazilian green propolis treatment in the prefrontal cortex

Gene Symbol	Gene Accession	Value (Log_2_ Ratio)			Log Ratio ^1)^	*p*-value ^1)^	Log Ratio ^2)^	*p*-value ^2)^
		Sham	Aβ	Aβ + PP900				
*Trem2*	NM_001272078	6.8	7.8	6.9	0.97	0.011	‒0.85	0.019
*Gbp3*	NM_001289492	5.2	6.0	5.6	0.87	0.00	‒0.44	0.036

^1)^ Log ratio between the sham and Aβ groups, *p*-value determined using Student’s *t*-test

^2)^ Log ratio between the Aβ and Aβ + PP900 groups, *p*-value determined using Student’s* t*-test

Sham group [sham operation plus vehicle (0.5% methylcellulose) treatment]; Aβ group (ICV Aβ_25‒35_ injection plus vehicle treatment); Aβ + PP900 group (ICV Aβ_25‒35_ injection plus 900 mg/kg b.w. Brazilian green propolis)

### Brazilian propolis prevented Aβ-induced Trem2 expression

**Fig. 4:**
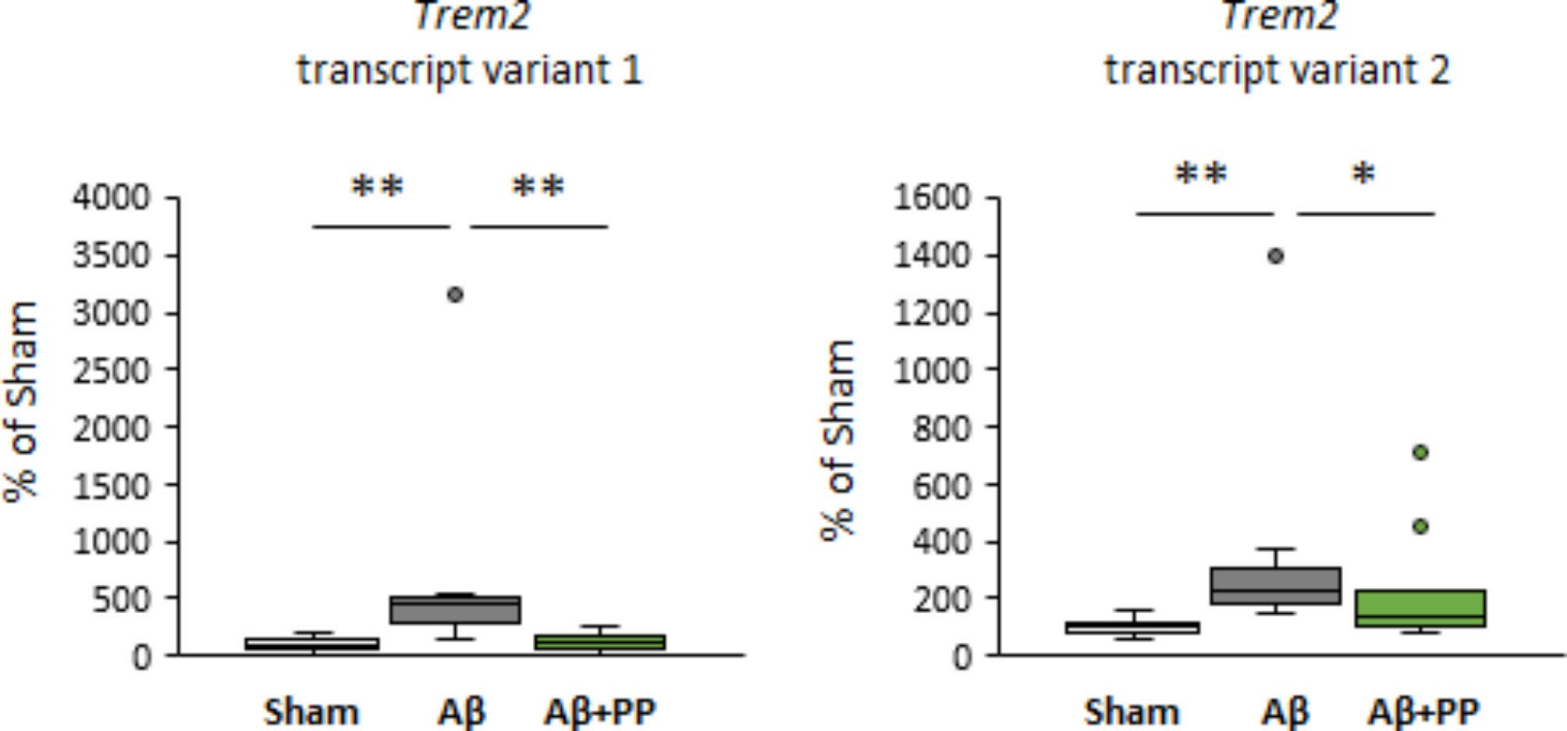
Quantitative evaluation of mRNA expression levels in the brain that changed in microarray analysis. The mRNA expression levels of *Trem2* transcript variants 1 and 2 in the hippocampus were measured using real-time reverse transcription polymerase chain reaction. Data were normalized with respect to a housekeeping gene, actin-β, and shown as boxplots (n = 8). Boxplots show minimum, first-quartile, median, third-quartile, and maximum values. Outliers are shown as circles. *P*-values were determined using one-way analysis of variance (post-hoc test Tukey’s test), * *P* < 0.05, ** *P* < 0.01

**Fig. 5:**
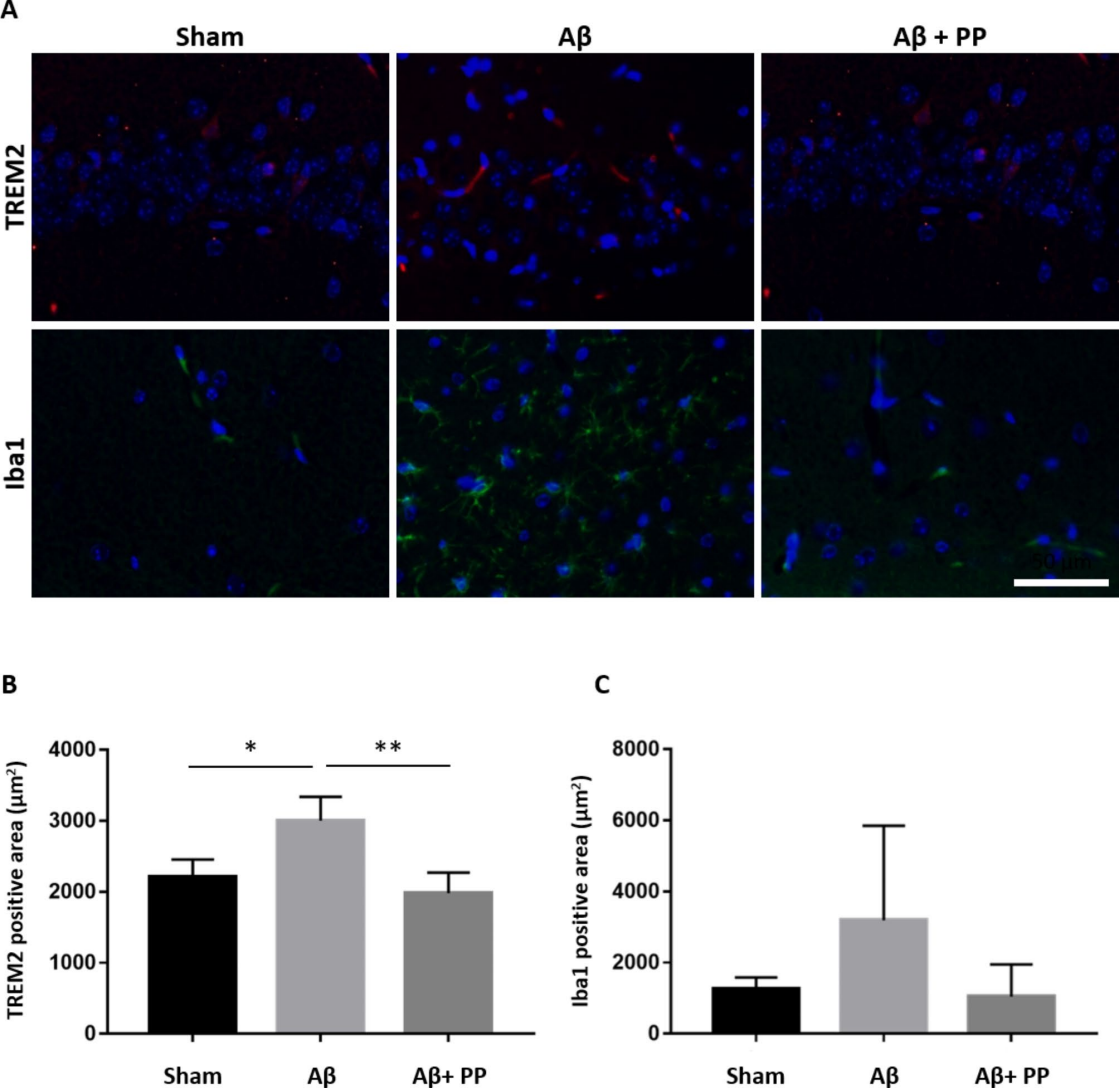
Effect of propolis on the TREM2 expression in the hippocampus induced by Aβ_25‒35_ injection. Immunofluorescence of TREM2 (**A**, upper panels) and IBA1 (**A**, lower panels) in the hippocampal CA1 of each group. Scale bars, 100 μm. Quantification of fluorescence intensity of TREM2 (**B**) and IBA1 (**C**) in the hippocampal CA1 of each group. Data are shown as mean ± standard error of mean. *P*-values are determined using one-way analysis of variance (post-hoc test Tukey’s test), * *P* < 0.05, ** *P* < 0.01

We further investigated the expression of TREM2, a receptor expressed on microglia, which is involved in immune and inflammatory responses in the brain. In the steady state, TREM2 is involved in neuromodulation through synaptic elimination by regulating the phagocytic capacity of the microglia. In contrast, *Trem2* expression is upregulated in the microglia of patients with AD, and its genetic polymorphisms are associated with AD pathology. We performed RT-qPCR to confirm *Trem2* expression which was altered in, both, the hippocampus and prefrontal cortex. Both the known *Trem2* transcripts were considerably induced by ICV Aβ injection. Thus, we confirmed that Brazilian green propolis treatment significantly suppressed Aβ-induced *Trem2* expression (Fig. 4). Further, we examined the microglial activation and neuroinflammation using immunofluorescent staining in the hippocampal CA1 sections of mice. The ICV injection of Aβ_25‒35_ significantly activated microglia in the test groups compared with that in the sham group, suggesting enhanced TREM2 expression (Fig. 5). No difference was observed in IBA1 expression among the test groups compared with that in the Aβ group. However, the Aβ + PP900 group showed a significant increase in TREM2 positive area at the same level as the sham group compared with the Aβ group (Fig. 5). Thus, pretreatment with Brazilian green propolis suppressed Aβ-induced microglia activation.

### Brazilian green propolis prevented Aβ-induced systemic inflammation

**Fig. 6:**
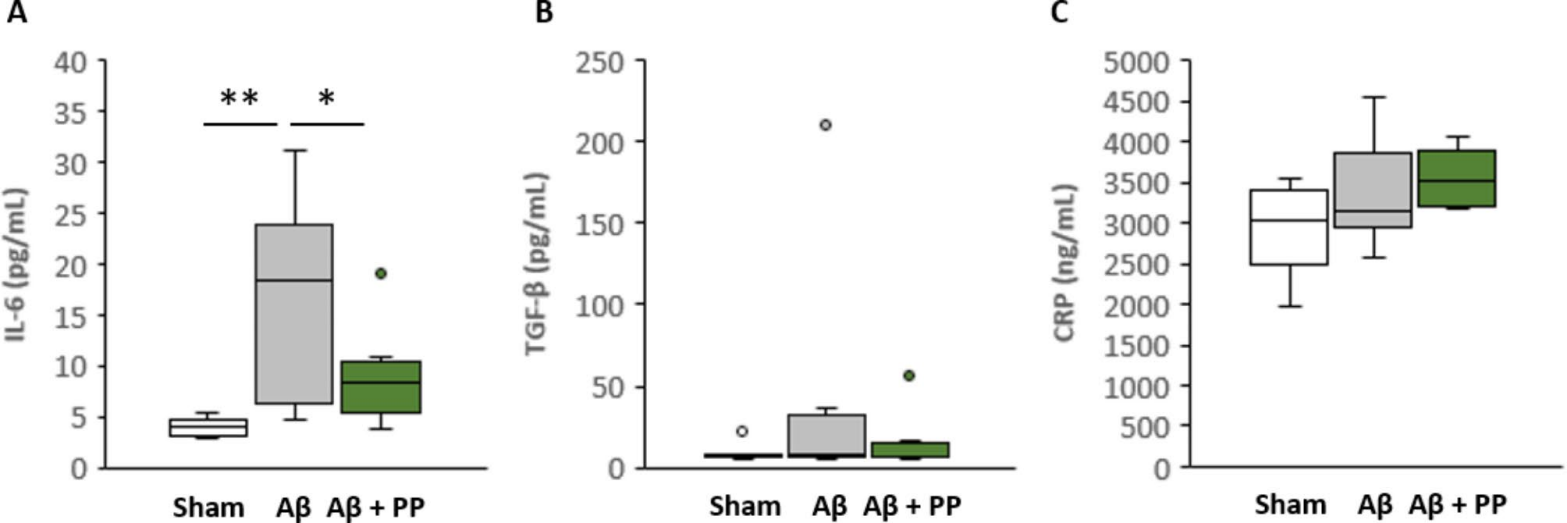
Effect of propolis on plasma cytokine levels. Interleukin-6 (**A**), transforming growth factor-β (**B**), and C-reactive protein (**C**) levels are shown as boxplots (n = 8). Boxplots show minimum, first-quartile, median, third-quartile, and maximum values. Outliers are shown as circles. *P*-values were determined using one-way analysis of variance (post-hoc test Tukey’s test), * *P* < 0.05, ** *P* < 0.01

The association between AD and systemic inflammation has been reported [34]. A clinical study has shown that Brazilian green propolis reverses cognitive decline along with reducing systemic inflammation in the older population living at high altitudes [11]. Therefore, we analyzed the effect of Brazilian green propolis on systemic inflammation using an ICV injection of Aβ_25‒35_. We measured the plasma levels of the inflammatory markers IL-6, TGFβ, and CRP, which are known to increase in patients with AD [35–37]. Plasma IL-6 levels significantly increased after ICV Aβ_25‒35_ injection (Fig. 6A), whereas plasma TGFβ and CRP levels remained unchanged (Fig. 6B–C). In contrast, combining these results with the microarray data confirmed that the expressions of these cytokines remained unchanged in both the hippocampus and prefrontal cortex. Thus, ICV injection of Aβ_25‒35_ partially contributed to systemic inflammation. We further investigated the effect of 900 mg/kg Brazilian green propolis administration on the levels of these inflammatory markers. As a result, Aβ_25‒35_-induced changes in IL-6 levels were significantly alleviated by Brazilian green propolis treatment. These results suggest that Brazilian green propolis partially prevented systemic inflammation induced by Aβ_25‒35_.

## Discussion

The present study aimed to elucidate the preventive effect of Brazilian green propolis on learning and memory impairment in AD model mice induced by Aβ_25‒35_ ICV infusion. This effect is attributed to the suppression of excessive immune and inflammatory responses in the brain induced by Aβ_25‒35_. In addition, Brazilian green propolis was found to suppress the systemic inflammation elicited by brain inflammation induced by Aβ_25‒35_.

The histological features of patients with AD are extracellular Aβ plaques and the accumulation of brain immune and inflammatory cells, such as microglia and astrocytes, around the Aβ plaques [24]. The expressions of immune and inflammatory response genes increase in the brains of patients with AD and AD model mice [22, 38, 39]. Cognitive decline in patients with AD is attributed to the excessive immune and inflammatory responses in the brain, as the depletion of glial cells improves learning and memory functions [39]. In this study, comprehensive gene expression analysis using microarrays revealed that ICV injection of Aβ_25‒35_ promoted immune and inflammatory responses in the hippocampus and prefrontal cortex (Fig. 3). Some genes were upregulated in activated microglia or astrocytes. In summary, these results suggest that ICV injection of Aβ_25‒35_ induced the activation of excessive immune and inflammatory responses in the brain, causing neurodegeneration and neurotransmission disorders, as well as learning and memory impairment in patients with AD.

Among the genes attenuated by Brazilian green propolis treatment (Tables 1 and 2), *Trem2* induction was suppressed by Aβ_25‒35_ in both the hippocampus and prefrontal cortex. Patients with AD have high TREM2 expression in the brain, and its genetic polymorphisms are associated with AD pathology. Additionally, TREM2 is a receptor for Aβ [27], and its signaling promotes Aβ clearance and inflammation response [26]. Animal models have shown that *Trem2*-knockout mice exacerbate cognitive function by reducing Aβ clearance [40]. In the present study, ICV injection of Aβ_25‒35_ significantly induced *Trem2* mRNA expression in the brain (Fig. 4). This presumably reflects the excessive activation of microglia to remove Aβ from the brain. Conversely, Brazilian green propolis treatment showed significant improvement in Aβ_25‒35_-induced increase in *Trem2* expression. These results suggest that prophylactic propolis treatment enhanced Aβ clearance capacity by promoting microglial phagocytosis and inhibited the excessive microglial activation in the brain induced by Aβ injection.

Lipocalin-2 (LCN2) showed maximum upregulation by Aβ_25‒35_ injection in the hippocampus and prefrontal cortex. LCN2, a 25-kDa secreted glycoprotein, is an acute-phase protein involved in iron metabolism [28]. LCN2 is upregulated in astrocytes and choroid plexus epithelial cells (CPECs) in the brain in response to Aβ_1–42_ [28, 29]. In LCN2-knockout mice, Aβ-induced death of astrocytes and CPECs is not induced. In the present study, Brazilian green propolis treatment inhibited the increase in LCN2 expression. These results suggest that the downregulation of LCN2 by Brazilian green propolis administration protected the brain from CPEC and astrocyte cell death, thereby improving cognitive function. In the hippocampus, Spp1, also called osteopontin, was suppressed by Brazilian green propolis treatment. The expression of *Spp1* is upregulated in the microglia of AD model mice [41]. CXCL13 is a B-lymphocyte chemotaxis factor and contributes to an inflammatory response by activating astrocytes via CXCR5 in a neuralgia model [42, 43], but there are no reports of AD-type dementia. We identified two downregulated genes, *Kcnj13* and *Folr1*, in the hippocampus after ICV Aβ_25‒35_ injection. As the functions of these genes in cognitive dysfunction remain unexplained, further studies are required to elucidate the role of these genes. Collectively, these results suggest that propolis protects against neurodegeneration and neural network disruption by suppressing Aβ-induced overactivation of immune and inflammatory cells, such as microglia and astrocytes, in the brain.

Propolis is a natural product comprising several compounds, including cinnamic acid derivatives and flavonoids [44]. Artepllin C, a cinnamic acid derivative, is the most abundant compound in Brazilian green propolis; it has antioxidant properties and inhibits neurodegeneration in vitro [8]. In addition, it contributes to synaptic efficacy by increasing the expressions of *BDNF* and *Arc* [8]. Kaemferide, the major flavonoid in Brazilian green propolis, activates transcriptional factors such as peroxisome proliferator-activated receptor alpha (PPARα) [44] and cAMP response element binding protein (CREB) [45]. PPARα agonist increases Aβ clearance in AD model mice [46]. CREB is involved in neurotransmission in the brain through neurotrophic factor expression [45]. These results suggested that multiple compounds in the propolis synergistically contribute to the anti-cognitive function observed in this study.

Systemic inflammation is one of the risk factors for AD [47, 48]. We demonstrated that Brazilian green propolis prevented plasma IL-6 elevation induced by ICV Aβ_25‒35_ injection. This finding suggests that propolis suppressed systemic inflammation derived from peripheral cells and tissues. There are some possible mechanisms for the interaction of inflammation between the central nerve system and peripheral tissues. First, the inflammation of the central nerve system is bridged to peripheral tissue or cells via the vagus nerve [49, 50]. One recent report suggested that ICV Aβ_1‒42_ injection induced gut microbiota dysbiosis and led to gut inflammation, via inhibiting the cholinergic anti-inflammatory pathway in the brain [51]. The possible second mechanism is the crosstalk between microglia and peripheral immune and inflammatory cells related to the systemic inflammation progression [34]. The activation of inflammatory cells is suppressed by treatment with Brazilian green propolis in several animal models [32, 52–55]. Therefore, the increase in plasma IL-6, observed in the Aβ_25−35_ ICV group, might be caused by the interaction between the brain and peripheral tissues. These results suggest that prophylactic treatment using Brazilian green propolis modulated the inflammatory states of these cells, suppressing plasma IL-6 levels.

In this study, we demonstrated the improvement of learning and memory impairment of Brazilian green propolis. However, there are some research limitations. The first is about AD-type dementia model mice. This study used an ICV injection of Aβ_25−35_ as an AD model. This model is suitable for short-term evaluation of cognitive decline due to the induction of oxidative stress, inflammations, and neurological disorders with Aβ_25−35_. In recent years, amyloid precursor protein knock-in mice have been developed, which mimic the onset of AD in humans [18]. The second is about the cognitive functional tests. In this study, we used a passive avoidance test suitable for evaluating learning and memory to investigate whether propolis prevents cognitive decline in the AD model. Multiple cognitive functional tests are required to fully elucidate the prevention of cognitive function with Aβ, such as the Y maze test, which evaluates spontaneous alternation behavior [20]; the Morris water maze test, which evaluates spatial memory [10]; and the novel object recognition test, which evaluates visual recognition memory [56]. Further research using these model systems is required to clarify the preventive effect of propolis on the cognitive decline. The third point is about the mechanism of action of propolis. In this study, we demonstrated that propolis suppresses inflammation in the brain and peripheral areas to prevent cognitive decline. However, the tissue distribution of the main components of propolis could not be demonstrated. It will be necessary to verify whether the propolis components reach the brain using imaging mass spectrometry and microdialysis.

## Conclusions

In the present study, we showed that Brazilian green propolis attenuated Aβ-induced memory and learning impairment. Comprehensive gene expression analysis demonstrated that Brazilian green propolis treatment suppressed inflammation and immune responses via immune cells such as microglia and astrocytes in the brain. These results indicate the potential of Brazilian green propolis as a promising ingredient for preventing AD-type dementia.

### Electronic supplementary material

Below is the link to the electronic supplementary material.

**Supplementary Material 1: Additional Table 1**. Upregulated genes induced by intracerebroventricular Aβ25?35 injection in the hippocampus. **Additional Table 2**. Downregulated genes induced by intracerebroventricular Aβ25?35 injection in the hippocampus. **Additional Table 3**. Genes with altered expressions induced by intracerebroventricular Aβ25?35 injection in the prefrontal cortex.

## Data Availability

The datasets in the current study available from the corresponding author on reasonable request.

## List of abbreviations

AD: Alzheimer’s disease
Aβ: Amyloid beta
CPECs: Choroid plexus epithelial cells
CRP: C-reactive protein
Ig: Immunoglobulin
IL-6: Interleukin 6
ICV: Intracerebroventricular
LCN2: Lipocalin-2
T-PBS: Phosphate-buffered saline with Tween 20
RT-qPCR: Quantitative real-time polymerase chain reaction
ss: Single-strand
TGFβ1: Transforming growth factor-β1
