# Supplementary material for: Brazilian green propolis prevent Alzheimer’s disease-like cognitive impairment induced by amyloid beta in mice
Source: BMC Complement Med Ther. 2023 Nov 17;23:416. doi: 10.1186/s12906-023-04247-7 (PMC10656927; doi:10.1186/s12906-023-04247-7)
Supplement: Supplementary file 1 — Supplementary Material 1: Additional Table 1. Upregulated genes induced by intracerebroventricular Aβ25?35 injection in the hippocampus. Additional Table 2. Downregulated genes induced by intracerebroventricular Aβ25?35 injection in the hippocampus. Additional Table 3. Genes with altered expressions induced by intracerebroventricular Aβ25?35 injection in the prefrontal cortex. [file 12906_2023_4247_MOESM1_ESM.docx]

**Additional information**

Additional Table 1. Upregulated genes induced by intracerebroventricular Aβ_25‒35_ injection in the hippocampus.

Additional Table 2. Downregulated genes induced by intracerebroventricular Aβ_25‒35_ injection in the hippocampus.

Additional Table 3. Genes with altered expressions induced by intracerebroventricular Aβ_25‒35_ injection in the prefrontal cortex.

**Additional Table 1. Upregulated genes with changed expressions induced by intracerebroventricular amyloid beta (Aβ)_25‒35_ injection in the hippocampus**

| **Gene Symbol** | **Gene Accession** | **Value (Log_2_ Ratio)** | | | **Log Ratio ^1)^** | **Log Ratio ^2)^** |
| --- | --- | --- | --- | --- | --- | --- |
|  |  | **Sham** | **Aβ** | **Aβ+PP900** |  |  |
| *Lcn2* | NM_008491 | 3.8 | 8.0 | 6.2 | 4.13 | ‒1.73 |
| *Mpeg1* | NM_010821 | 7.5 | 10.4 | 9.8 | 2.93 | ‒0.63 |
| *Spp1* | NM_001204201 | 5.2 | 8.0 | 6.9 | 2.84 | ‒1.15 |
| *Ccl3* | NM_011337 | 4.7 | 7.0 | 6.4 | 2.29 | ‒0.57 |
| *Trem2* | NM_001272078 | 6.5 | 8.7 | 7.4 | 2.20 | ‒1.29 |

^1)^ Log ratio between the sham and Aβ groups

^2)^ Log ratio between the Aβ and Aβ+PP900 groups

**Additional Table 2. Downregulated genes with changed expressions induced by intracerebroventricular Aβ_25‒35_ injection in the hippocampus.**

| **Gene Symbol** | **Gene Accession** | **Fold Change (Log_2_ Ratio)** | | | **Log Ratio ^1)^** | **Log Ratio ^2)^** |
| --- | --- | --- | --- | --- | --- | --- |
|  |  | **Sham** | **Aβ** | **Aβ+PP900** |  |  |
| *Kcnj13* | NM_001110227 | 14.0 | 10.1 | 11.08 | −3.95 | 1.02 |
| *Folr1* | NM_001252552 | 11.4 | 8.1 | 9.20 | −3.29 | 1.09 |
| *Clic6* | NM_172469 | 13.4 | 10.4 | 11.30 | −3.00 | 0.90 |
| *Kcne2* | NM_134110 | 8.9 | 6.4 | 7.17 | −2.44 | 0.75 |

^1)^ Log ratio between the sham and Aβ groups

^2)^ Log ratio between the Aβ and Aβ+PP900 groups

**Additional Table 3. Genes with altered expressions induced by intracerebroventricular Aβ_25‒35_ injection in the prefrontal cortex.**

| **Gene Symbol** | **Gene Accession** | **Value (Log_2_ Ratio)** | | | **Log Ratio ^1)^** | **Log Ratio ^2)^** |
| --- | --- | --- | --- | --- | --- | --- |
|  |  | **Sham** | **Aβ** | **Aβ+PP900** |  |  |
| *Lcn2* | NM_008491 | 3.6 | 5.8 | 4.8 | 2.25 | −0.96 |
| *Gfap* | NM_001131020 | 10.3 | 12.0 | 11.8 | 1.69 | −0.20 |

^1)^ Log ratio between the sham and Aβ groups

^2)^ Log ratio between the Aβ and Aβ+PP900 groups
